# Association between active commuting and elevated blood pressure in adolescents

**DOI:** 10.1590/S1679-45082017AO4093

**Published:** 2017

**Authors:** Fábio da Silva Santana, Aline Cabral Palmeira, Marcos André Moura dos Santos, Breno Quintella Farah, Bruna Cadengue Coêlho de Souza, Raphael Mendes Ritti-Dias

**Affiliations:** 1 Faculdade de Comunicação Tecnologia e Turismo de Olinda, Olinda, PE, Brazil.; 2 Universidade de Pernambuco, Petrolina, PE, Brazil.

**Keywords:** Motor activity, Exercise, Adolescent, Hypertension, Public health, Atividade motora, Exercício, Adolescente, Hipertensão, Saúde pública

## Abstract

**Objective:**

To analyze the association between active commuting and blood pressure in adolescents.

**Methods:**

This is a cross-sectional study with high school students from public education network in the state of Pernambuco, Brazil. Data from 6039 students (14 to 19 years) were collected using a questionnaire. “Physically inactive” were considered those who reported not to walk or ride a bicycle to and from school on any day of the past week, and/or those who, regardless of the weekly frequency of practice this type of activity, reported the duration of commuting to school was less than 20 minutes (round trip). The high blood pressure was obtained by Omron HEM 742 equipment. Adolescents with high blood pressure were defined as those with higher blood pressure or equal to the 95th percentile for age, sex and height. Regression logistic analyses were used to assess the association between active commuting and high blood pressure, considering adjustments for the following confounders: sex, age, overweight, total physical activity, socioeconomic level, place of residence.

**Results:**

The prevalence of high blood pressure was 7.3%, and 79.3% were considered insufficiently active in commuting. There was an association between high blood pressure and active commuting only among those living in rural areas (OR = 6.498; 95% CI = 1.513-27.900), and the same was not observed among those living in urban areas (OR = 1.113; 95% CI = 0.812-1.526).

**Conclusion:**

Active commuting can be considered a protective factor for high blood pressure in adolescents living in rural areas.

## RESUMO

**Objetivo:**

Analisar a associação entre o deslocamento ativo e a pressão arterial elevada em adolescentes.

**Métodos:**

Trata-se de um estudo transversal com estudantes do Ensino Médio da rede pública estadual de Pernambuco. Os dados de 6.039 estudantes de 14 a 19 anos foram coletados mediante questionário. Foram considerados “insuficientemente ativos” aqueles que relataram que em nenhum dos dias da última semana realizaram deslocamentos a pé ou de bicicleta e/ou aqueles que, independentemente da frequência semanal de prática desta atividade, relataram que a duração do deslocamento para a escola era inferior a 20 minutos (ida e volta). A pressão arterial elevada foi obtida por meio do equipamento Omron HEM 742. Foram definidos como adolescentes com pressão arterial elevada aqueles com pressão arterial maior ou igual ao percentil 95 para idade, sexo e estatura. Recorreu-se à análise de regressão logística para verificar a associação entre deslocamento ativo e pressão arterial elevada, considerando ajustes para os fatores de confusão sexo, idade, sobrepeso, atividade física total, nível socioeconómico e zona de moradia.

**Resultados:**

A prevalência de pressão arterial elevada foi de 7,3% e de inatividade física no deslocamento foi de 79,3%. Foi verificada associação entre pressão arterial elevada e deslocamento ativo apenas entre aqueles que moram em zona rural (OR=6,498; IC95%=1,513-27,900). O mesmo não foi verificado entre aqueles que moravam na zona urbana (OR = 1,113; IC95%=0,812-1,526).

**Conclusão:**

O deslocamento ativo póde ser considerado fator de proteção para a pressão arterial elevada nos adolescentes que moravam na zona rural.

## INTRODUCTION

High blood pressure (BP) is one of the major risk factors for cardiovascular disease and, when present in childhood, it may be considered a predictor of cardiovascular mortality risk in adult life. This is important because some studies have shown that the prevalence of high BP in pediatric populations is high in developed and developing countries, ranging between 3.6 and 19.4%.^(^ ^1^ ^)^ In Brazilian adolescents, the prevalence of high BP was 8.75% in boys and 6.31% in girls.^(^ ^2^ ^)^

While the prevalence of high BP increases,^(^ ^3^ ^)^ there is a decrease in the level of physical activity in this population, as well as an increase in activities that require a longer time sitting.^(^ ^4^ ^)^ These behaviors lead to harmful health effects in adolescents, including decreased aerobic fitness and glucose tolerance.^(^ ^5^ ^)^ In this context, incorporating physical activity into one's lifestyle is one of the strategies used to reduce at-risk conditions, such as high BP.

Active commuting by walking and cycling has been associated to an overall increase in physical activities and improvement of population health indicators.^(^ ^6^ ^)^ Although there is evidence demonstrating the association between physical activity and BP,^(^ ^7^ ^)^ active commuting and decreased cardiovascular risk,^(^ ^8^ ^)^ few studies have looked into the specific contribution of active commuting to BP values. It is known that active commuting is associated with lower overweight and obesity rates among adolescents,^(^ ^9^ ^)^ and that said conditions contribute to increase BP.

In adults, systolic BP values are lower in individuals who are active during commuting time.^(^ ^10^ ^)^ In adolescents, however, only one study was found the association between active commuting and BP.^(^ ^8^ ^,^ ^11^ ^-^ ^13^ ^)^ Also, the reasons for these discrepancies are not yet clear, since the differences in intensity, volume and frequency of activity during commuting time were not assessed, and there are no standardized measurements for physical activity. Another fact that should be considered is that other factors, such as gender (boys seem to be more active than girls),^(^ ^14^ ^)^ place of residence (i.e. distance between home and school) and socioeconomic level, may influence the way adolescents commute.

There are no records in the literature of interventions using active commuting to decrease BP among adolescents, but only for adults with clinical conditions.^(^ ^15^ ^)^ Thus, understanding active commuting and its association with BP in adolescents could support the use of programs encouraging this practice among the young.

## OBJECTIVE

To investigate the association between active commuting and high blood pressure in adolescents.

## METHODS

A cross-sectional, school-based and statewide epidemiological study conducted in 2011. The data used in this study come from the project *Physical Activity and Health Risk Behaviors in High School Students in the State of Pernambuco: A Temporal Trend Study (ATITUDE Project 2006-2011).* It was approved by the Institutional Review Board of the *Universidade de Pernambuco,* under number 159/10, CAAE: 0158.0.097.000-10. All volunteers signed an Informed Consent. For subjects aged under 18 years, we adopted the Negative Consent Form so that parents and/or guardians could authorize the enrollment of their children in the study.

Since this is a subanalysis under the *Atitude Project*, whose target population were male and female adolescents s (14 to 19 year-old) enrolled in high school in the public education network of the state of Pernambuco, the sample calculation was the same as that of the project. To calculate the sample size, we considered the estimated population size of 338,698 adolescents; a 95% confidence interval; a 2% sampling error; estimated prevalence of 50%; a design effect *(deff)* of 2; and an additional margin of 20%, considering potential losses and refusals. The 50% prevalence was chosen because this study is part of a greater project aiming to investigate multiple health risk behaviors occurring at different frequencies.

This study excluded adolescents aged under 14 and over 19 years; those self-reporting diabetes, cardiovascular and/or neurological disease; those who failed to properly answer the questionnaire; and those who refused to undergo anthropometric and BP measurements. Thus, the final sample was composed of 6039 adolescents.

The data were collected between May and October 2011 at the time of day they attended classes (morning, afternoon, evening). Information on age, ethnicity, place of residence and level of physical activity were obtained through a questionnaire adapted from the Global School-Based Student Health Survey (GSSH), which was proposed by the World Health Organization and is commonly used in epidemiological studies.^(^ ^16^ ^,^ ^17^ ^)^ For this sample, the agreement analysis obtained from the retesting of a sub-sample presented Kappa values (agreement analysis using the test-retest with a one-week interval) of 0.59, for both questions.

To attest the active commuting, we used questions on the number of days and commuting time: “During the past 7 days, on how many days did you walk or ride a bicycle to and from school?” and “During the past 7 days, on average, how much time per day did you spend commuting to and from school (consider the total time of the round-trip to and from school)”.

We considered as “insufficiently active” those who reported not to walk or ride a bicycle to and from school on any day of the past week, and/or those who, irrespective of the weekly frequency of this type of activity, reported that the commuting time to and from school was under 20 minutes (round-trip).^(^ ^18^ ^)^

BP was measured using the Omron HEM 742 monitor (Omron, Shanghai, China).^(^ ^19^ ^)^ Before measurements were taken, the adolescents sat with their legs uncrossed for 5 minutes. The cuff used was appropriate for the size of each adolescent. Three measurements were taken on the right arm, with subjects in a seated position. For analysis, we used the average of the last two measurements. High BP was defined as systolic or diastolic pressure equal to or greater than the 95^th^ percentile, based on gender, age and height.^(^ ^20^ ^)^

Portable scales (Tanita, Brazil) and a stadiometer (Welmy, Brazil) were used to determine body mass and height. Both measurements were taken as per the procedures described above.^(^ ^21^ ^)^ Excess weight was determined by a body mass index above the 85^th^ percentile for the respective age.^(^ ^22^ ^)^ The level of total physical activity, socioeconomic level and type of residence were obtained from the questionnaire. The level of total physical activity was determined by the question: “In a typical or regular week, on how many days are you physically active for a total of at least 60 minutes a day?” Those who claimed to be active on 5 days or more were considered active. The socioeconomic level was identified by the maternal schooling level, and the subject with the highest socioeconomic level reported that their mother had over 8 years of education. The type of residence was determined by the answer to the following question: “Do you live in a rural or urban area?”

All statistical analyses were performed using the Statistical Package of the Social Sciences (SPSS), version 20 (IBM Corp, Armonk, New York). The level of physical activity was described as frequency, and the other measurements as mean ± standard deviation. Logistic regression analysis was used to investigate the association between high BP and level of activity while commuting for both genders. The model was adjusted for potential confounders, such as sex, age, overweight, total physical activity, socioeconomic level and type of residence.^(^ ^23^ ^)^ After adjusting for the different variables in each model, the odds ratio (OR) and the respective 95% confidence intervals (95% CI) were found significant for a p-value ≤0.05.

## RESULTS

A total of 6,039 adolescents were evaluated in this study, of which 3,633 were girls. The general characteristics of subjects are described in table 1.

**Table 1:** Characteristics of the study participants

General variables		Boys (n=2,406)	Girls (n=3,633)	p value
Weight, kg		61.6±13	53.1±12	<0.001
Height, cm		172±9	159±8	<0.001
Body mass index, kg/m^2^		20.8±3.9	20.8±4	0.895
Systolic blood pressure, mmHg		122±17	112±14	<0.001
Diastolic blood pressure, mmHg		67±11	68±11	<0.001
Type of residence, (%)				
	Urban	74.6	74.5	0.938
	Rural	25.4	25.5	
Socioeconomic level, (%)				
	Higher	87.7	84.9	0.003
	Lower	12.3	15.1	
Commuting time, (%)				
	<10 minutes	9.7	11.4	0.270
	10-20 minutes	2.8	2.8	
	>20 minutes	87.5	85.8	
Active commuting, days				
	None	43.5	41.5	
	1	2.6	2.9	0.311
	2	2.3	2.1	
	3	2.4	1.0	
	4	1.7	1.9	
	5	26.5	28.9	
	6	3.2	3.0	
	7	17.8	17.9	
Total physical activity, (%)				
	Active	61.8	72.5	0.258
	Inactive	38.2	27.5	

**Figure 1:**
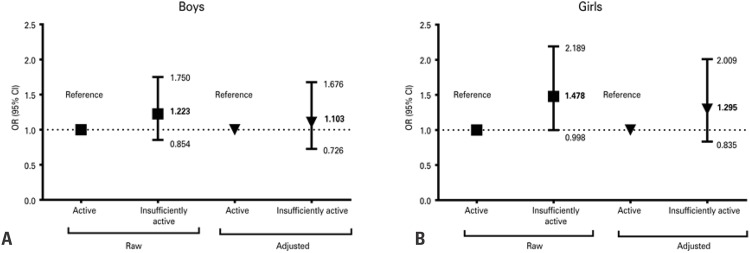
(A) Prevalence of insufficiently active (sum of variables days and commuting time) and (B) high blood pressure in adolescents

In the total sample the prevalence of high BP was 7.3%; and 79.3% of adolescents were insufficiently active during the commuting, *i.e.,* they did not walk or ride a bicycle to school on any day of the week or, if they did, they manage to do in less than 20 minutes. When assessed by sex, the prevalence of high BP was 10.4 and 5.2% for boys and girls, respectively. As for active commuting, the prevalence of insufficiently active commuting was 81.6% and 77.8% for boys and girls, respectively (Figure 1).

**Figure 2:**
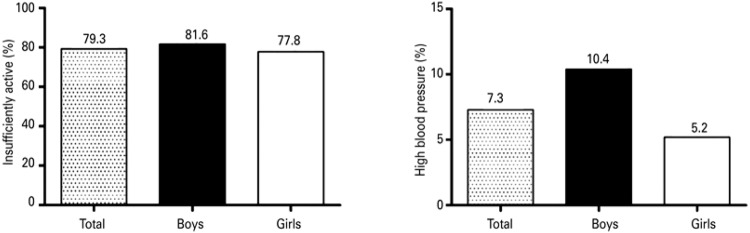
Association between high blood pressure and insufficiently active commuting. Model adjusted per age, overweight, total physical activity, socioeconomic level and type of residence OR: *odds ratio;* 95% CI: 95% confidence interval.

The results of the association, both raw and adjusted, stratified by sex are described in figure 2.

In both the raw and adjusted analyses, there was no association between high BP and active commuting in both sexes. When stratifying the sample based on type of residence (urban and rural), there was an association between high BP and insufficiently active commuting only among those with rural residence (OR=6.498, 95% CI=1.513-26.900) (Table 2).

**Table 2:** Association between high blood pressure and insufficiently active commuting. Model stratified by type of residence, and adjusted per sex, overweight, total physical activity, socioeconomic level, type of residence and age

Zone	Raw analyses		Adjusted analysis	
	OR	95% CI	OR	95% CI
Urban	0.716	0.580-0.882	1.113	0.812-1.526
Rural	1.397	1.133-1.723	6.498	1.513-27.900

OR: *odds ratio;* 95% CI: 95% confidence interval.

## DISCUSSION

The results found in the present study demonstrated that active commuting does not influence BP in both sexes; however there was an association between BP and active commuting among adolescents living in rural areas, where insufficiently active adolescents showed higher risk for high BP when compared to those physically active.

The prevalence of physical inactivity during the commuting to and from school was high when compared to the findings of a study with Danish adolescents,^(^ ^24^ ^)^ but lower than that verified in Americans.^(^ ^25^ ^)^ However, some aspects should be considered when comparing these prevalences, such as conditions that favor active commuting. The absence of cycle paths on the way to and from school, insufficient bike parking, the family's purchasing power, poor signage on the roads, and poor traffic safety can possibly explain the low prevalence of active commuting.^(^ ^26^ ^)^

In this study, the prevalence of high BP in the total sample was 7.3%, 10.4% for boys and 5.2% for girls. A systematic review study showed that the prevalence of high BP in Brazilian adolescents is 8.1%. In the northeast, the total prevalence was 11.2%; in that, 13.6% in boys and 8.5% in girls.^(^ ^2^ ^)^ The increase in obesity and high BP in adolescents can at least partly explain the increased prevalence found in this study, since obesity is closely related with the onset of hypertension.^(^ ^25^ ^)^

In the present study, there was an association between the type of residence and active commuting. Evidence suggests that, although rural adolescents are more active, in general,^(^ ^27^ ^)^ when the active commuting is further investigated, the result is the opposite - those living in rural areas being less prone to actively commuting to and from school^(^ ^28^ ^)^ as compared to those living in the city. This fact could be related to accessibility of public transportation and the distance from home to school. A cohort study carried out in two English cities showed that the distance from home to school has been increasing lately - from 1.6km, in 1975, to 2.3km, in 2001.^(^ ^29^ ^)^ This increase in the distance is likely associated with the fact that new schools are not built at the pace at which cities grow, which ends up stretching the distance from home to school and increasing the use of passive transportation.^(^ ^30^ ^)^

In practice, the results of this study suggest that encouraging active commuting among rural adolescents could lead to benefits regarding BP levels. One line of concern is that the prevalence of high BP in this study was twice as large in boys than in girls. Despite the growing number of research studies on physical activity, there are still many gaps in understanding active commuting and its effects on BP levels. Some factors, such as classification of level and duration of physical activity, energy intake, and the different ways used to commute may influence these results. These elements should be investigated in future studies.

This study has some limitations. Its cross-sectional design prevents establishing causality and does not assess the impact of changes on active commuting in rural and urban areas over time. The distance from home to school was not obtained, which might lead to an over or underestimation of the results. No information on the sexual maturation stage was requested and, therefore, the impact of biological maturation on the results could not be controlled. BP was measured at one single timepoint, and safety-related aspects were not obtained. However, one advantage of this measurement is that it makes it possible to determine the frequency of chronic diseases and point out probable risk factors. Still, the costs are relatively lower relative to other types of studies.

Some strengths of this study should be stressed, such as the sample size, which establishes a representative analysis of the adolescent population, and the sampling procedures, which allowed for a sample of both boys and girls, living in different locations in the rural and urban areas of the state of Pernambuco.

## CONCLUSION

Active commuting seems to be a protective factor for high blood pressure in rural adolescents.
